# Does anonymity increase the reporting of mental health symptoms?

**DOI:** 10.1186/1471-2458-12-797

**Published:** 2012-09-17

**Authors:** Nicola T Fear, Rachel Seddon, Norman Jones, Neil Greenberg, Simon Wessely

**Affiliations:** 1 Academic Centre for Defence Mental Health, King’s College London, Weston Education Centre, 10 Cutcombe Road, London SE5 9RJ, UK

**Keywords:** Military, Mental health, Anonymity, Stigma, Barriers to care

## Abstract

**Background:**

There is no doubt that the perceived stigma of having a mental disorder acts as a barrier to help seeking. It is possible that personnel may be reluctant to admit to symptoms suggestive of poor mental health when such data can be linked to them, even if their personal details are only used to help them access further care. This may be particularly relevant because individuals who have a mental health problem are more likely to experience barriers to care and hold stigmatizing beliefs. If that is the case, then mental health screening programmers may not be effective in detecting those most in need of care. We aimed to compare mental health symptom reporting when using an anonymous versus identifiable questionnaire among UK military personnel on deployment in Iraq.

**Methods:**

Survey among UK military personnel using two questionnaires, one was anonymous (n = 315) and one collected contact details (i.e. identifiable, n = 296). Distribution was by alternate allocation. Data were collected in Iraq during January-February 2009.

**Results:**

No significant difference in the reporting of symptoms of common mental disorders was found (18.1% of identifiable vs. 22.9% of anonymous participants). UK military personnel were more likely to report sub-threshold and probable PTSD when completing questionnaires anonymously (sub-threshold PTSD: 2.4% of identifiable vs. 5.8% of anonymous participants; probable PTSD: 1.7% of identifiable vs. 4.8% of anonymous participants). Of the 11 barriers to care and perceived social stigma statements considered, those completing the anonymous questionnaire compared to those completing the identifiable questionnaire were more likely to endorse three statements: “leaders discourage the use of mental health services” (9.3% vs. 4.6%), “it would be too embarrassing” (41.6% vs. 32.5%) and “I would be seen as weak” (46.6% vs. 34.2%).

**Conclusions:**

We found a significant effect on the reporting of sub-threshold and probable PTSD and certain stigmatizing beliefs (but not common mental disorders) when using an anonymous compared to identifiable questionnaire, with the anonymous questionnaire resulting in a higher prevalence of PTSD and increased reporting of three stigmatizing beliefs. This has implications for the conduct of mental health screening and research in the US and UK military.

## Background

The US Department of Defense is currently carrying out a large mental health screening programme with the aim of detecting mental health disorders in military personnel on their return from Iraq or Afghanistan, the Post-Deployment Health Assessment (PDHA) and Post-Deployment Health Re-Assessment (PDHRA) [1]. The PDHA and the PDHRA use brief screening instruments which are administrated post-deployment to identify those individuals who require referral to mental health professionals and thus data collection is not anonymous. However, the effectiveness of these programmes remains in doubt. Milliken et al [1] found that not everyone identified via these programmes went on to attend a mental health clinic and that many of those who did attend mental health clinics did so in spite of not being identified as being in need of care by the screening process. Also Warner et al [2] reported that the PDHA misses most soldiers with mental health problems.

There is no doubt that the perceived social stigma of having a mental disorder acts as a substantial barrier to help seeking in both the UK and US Armed Forces [2-5]. It is possible that personnel may be reluctant to admit to symptoms suggestive of poor mental health when such data can be linked to them personally, even if their personal details are only used to help them access further care. This may be particularly relevant because individuals who have a mental health problem are more likely to report barriers to care and hold stigmatizing beliefs [2,4,5]. It is well documented that an individual’s beliefs about how they will be perceived by others if they have a mental health problem are powerful determinants of help-seeking [6]. If that is the case, then mental health screening programmers may not be effective in detecting those most in need of care.

The aim of this paper was to examine the reporting of mental health problems using an identifiable and an anonymous questionnaire completed by UK military personnel. Data were collected during deployment to Iraq (in early 2009) as part of an in-theatre mental health survey (Operational Mental Health Needs Evaluation, OMHNE) [7].

## Method

The OMHNE visit was conducted at various locations in Iraq between 26 January and 27 February 2009, with the research team consisting of military and civilian personnel [7]. True random sampling was not possible because of the need to ensure an adequate coverage of personnel from small widely dispersed operating bases, and for operational reasons. Instead, after discussion with the medical and personnel staff officers based in the UK operational headquarters, purposive sampling was conducted to ensure an adequate spread of personnel and locations. Information on the service and rank profile of the deployed force was obtained from the divisional personnel report, allowing examination of the representativeness of the study sample. All the main UK bases in Iraq were visited, excluding some remote locations. Location commanders were asked to assemble all available personnel so that the survey team could provide information about the study and distribute questionnaires. The survey team were explicit in briefing the potential respondents that, unlike other deployment activities, completion of the questionnaires was voluntary. Personnel were also assured that all information was confidential, that their individual responses would not be reported to commanders and that no individual would be named in any report about the study. Respondents were informed that any personal identification information would be separated from the questionnaire by the study team and stored separately. The questionnaire took approximately 25 minutes to complete. Participants were not given any payment or any other inducement for taking part in the study. Once completed, participants placed their questionnaire in an envelope and sealed it before giving it to a member of the study team.

The study used two versions of the OMHNE questionnaire. One was completely anonymous in that it asked for no information which could be used to identify the respondent. The other version asked for a variety of identifiable information, including: first name, surname, date of birth, Service number, and both unit and home address. Approximately 50% of the participants filled in anonymous questionnaires, with the other 50% being asked to provide contact details. The operational environment mandated a simple system of allocation, so we used alternate allocation to distribute the questionnaires. The questionnaires were distributed by the study team directly to the individuals and thus every other questionnaire allocated was anonymous. Personnel were reassured that no matter which version of the questionnaire they received no identifiable information would be released by the research team to anyone (including the Chain of Command) and that no participant would be referred on for care. All participants were UK military personnel on operational deployment to Iraq and were a non-help seeking group. Special Forces personnel were not included in the sample. Full details of the study methodology can be found in Mulligan et al [7].

### Measures used

#### Mental health measures

The OMHNE questionnaire included two mental health measures. The 12-item General Health Questionnaire (GHQ), which was used to measure symptoms of common mental disorders [8]. The GHQ asks participants to rate their health according to each of 12 items in relation to the last few weeks (compared to usual). Response categories are scored from 0-3 and were re-coded into binary scores (0-1) such that the responses ‘better than usual’ and ‘same as/no more than usual’ were coded 0, and the responses ‘rather more than usual’ and ‘much more than usual’ were coded as 1. The responses are summed and a caseness cut off score of ≥4 was used to define having symptoms of common mental disorder [8].

The Post-Traumatic Stress Disorder (PTSD) Checklist Civilian Version (PCL-C) was used to measure probable PTSD [9], with a score of ≥50 being used to define those with probable PTSD [9]. We have examined the PCL score in four categories to aid interpretation (17-19, 30-39, 40-49 (defined as sub-threshold PTSD) and 50+ (defined as probable PTSD). We also examined the three domains of PTSD, using the definition of one positive endorsement for intrusiveness (criterion B), three endorsements for numbing/avoidance (criterion C) and two endorsements for hyper-arousal (criterion D).

#### Stigmatizing beliefs

The OMHNE questionnaire included a list of 11 stigma statements (adapted from Hoge et al [3]). These were five statements referring to barriers to care, for example, “it’s difficult to schedule an appointment”, and six statements referring to perceived social stigma, for example, “I would be seen as weak”. Response options were on a 4-point Likert scale from strongly agree to strongly disagree. For the purposes of these analyses, responses were combined to form two response options (agree vs. disagree).

#### Socio-demographic, military and deployment factors

The questionnaire included questions about socio-demographic and military characteristics and deployment experiences. Combat exposure was assessed with a 17-item measure that asked about the frequency of exposure to potentially traumatic combat events. For each combat exposure, respondents were asked how many times in their current deployment they had experienced this exposure (never, once, 2-4 times, 5-9 times or 10+ times). This measure was adapted from Hoge et al [3]. For the purposes of the current analyses, exposure to each combat experience was summed and then the overall distribution was divided into tertiles (no exposures, 1-2 or 3+).

#### Ethical issues

Approval to conduct the study was granted by the Ministry of Defence Research Ethics Committee (MODREC) (Protocol No. 839/194). All participants gave written informed consent. The study complies fully with the Helsinki Declaration.

#### Analysis

Chi^2^ and *t*-test statistics were used to compare the categorical and continuous socio-demographic, military and deployment characteristics of the two groups. Chi^2^ test statistics and odds ratios (with 95% confidence intervals) were used to determine whether completing an identifiable or anonymous questionnaire influenced the likelihood of reporting mental health symptoms and stigmatizing beliefs. The data were analyzed using STATA 11, with statistical significance defined as P < 0.05.

## Results

Of 612 personnel approached to take part, 611 (99.8%) completed the survey. This represented approximately 15% of the UK Armed Forces personnel deployed to Iraq at the time the data were collected. The sample consisted of Naval Service (including Royal Marines), British Army and Royal Air Force personnel, men, women, regular and reserve personnel. The overall prevalence of probable PTSD was 3.3% (20/605) and 20.5% (121/590) for symptoms of common mental disorders. Of the 20 cases with probable PTSD, 16 also reported symptoms of common mental disorders.

296 (48.4%) respondents completed the identifiable questionnaire and 315 (51.6%) respondents completed the anonymous questionnaire. The socio-demographic, military and deployment characteristics of the two groups were similar (Table 1). There was one exception, Service, where there were proportionately more participants from the Army in the identifiable group. Also noteworthy is that in the anonymous group, 16 participants did not report whether they were male or female.

**Table 1:** Socio-demographic, military and deployment characteristics by questionnaire type (Anonymous vs. Identifiable)

	**Identifiable (n = 296)**	**Anonymous (n = 315)**	**Chi**^**2**^**or *t*-test statistic (degrees of freedom), P value**
	**n (%)**	**n (%)**	
**Sex**			
Men	266 (89.9)	262 (87.6)	0.75 (1), P = 0.388
Women	30 (10.1)	37 (12.4)	
Missing data	-	16	
**Mean age in years**	27.9	27.6	0.48 (597), P = 0.631
(95% confidence interval)	(27.0-28.7)	(26.7-28.4)	
Missing data	3	9	
**Enlistment type**			
Regular	281 (94.9)	296 (94.0)	0.27 (1), P = 0.603
Reserve	15 (5.1)	19 (6.0)	
**Service**			
Naval Service	10 (3.4)	29 (9.2)	8.69 (2), P = 0.013
Army	249 (84.1)	248 (78.7)	
Royal Air Force	37 (12.5)	38 (12.1)	
**Rank**			
Junior	208 (70.5)	223 (71.0)	0.14 (2), P = 0.932
Senior	51 (17.3)	51 (16.2)	
Officer	36 (12.2)	40 (12.7)	
Missing data	1	1	
**Thought might be killed**			
No	204 (69.9)	223 (73.1)	0.77 (1), P = 0.379
Yes	88 (30.1)	82 (26.9)	
Missing data	4	10	
Number of traumatic events experienced*			
None	103 (35.0)	99 (31.5)	1.94 (2), P = 0.380
1-2	86 (29.3)	108 (34.4)	
3+	105 (35.7)	107 (34.1)	
Missing data	2	1	

*Sum of number of times experienced a range of combat related events.

There was no statistically significant difference in the reporting of symptoms of common mental disorders by method of data collection. However, respondents who completed anonymous questionnaires were significantly more likely to be defined as having sub-threshold and probable PTSD than those who completed identifiable questionnaires (Table 2). These differences resulted in significantly raised odds ratios, which remained significant following adjustment for Service. Despite this difference, the prevalence of probable PTSD (and thus the number of individuals affected) within the anonymous group remains low (4.8% in the anonymous group (n = 15) vs. 1.7% in the identifiable group (n = 5)). We also examined the difference in reporting of the three PTSD domains, all three were more likely to be endorsed for those completing the anonymous vs. identifiable questionnaires (domain B (intrusiveness): P = 0.042; domain C (numbing/avoidance): P = 0.001; domain D (hyper-arousal): P = 0.036) (data not shown, but available from the authors).

**Table 2:** Questionnaire Type (Anonymous vs. Identifiable) and Symptom Reporting

	**Identifiable (n = 296)**	**Anonymous (n = 315)**	**Chi**^**2**^**test statistic (degrees of freedom), P value**	**Odds ratio (95% confidence interval)**	Adjusted* odds ratio (95% confidence interval)
	**n (%)**	**n (%)**			
PTSD (score on the PCL)					
- 17-29	260 (88.4)	253 (81.4)	9.66 (3), P = 0.022	1.00	1.00
- 30-39	22 (7.5)	25 (8.0)		1.17 (0.64-2.12)	1.20 (0.66-2.19)
- 40-49 (sub-threshold PTSD)	7 (2.4)	18 (5.8)		2.64 (1.09-6.44)	2.74 (1.12-6.69)
- 50+ (probable PTSD)	5 (1.7)	15 (4.8)		3.08 (1.10-8.61)	3.18 (1.13-8.90)
Missing data	2	4			
Common mental disorders					
- case (cut off 4+)	52 (18.1)	69 (22.9)	2.08 (1), P = 0.150	1.34 (0.90-2.01)	1.43 (0.95-2.14)
Missing data	8	13			

*Adjusted for Service.

Table 3 reports the barriers to care and perceived social stigma held by the two groups. Those completing the anonymous questionnaire were more likely than those completing the identifiable questionnaire to endorse three of the 11 stigmatizing beliefs: “leaders discourage the use of mental health services” (9.3% vs. 4.6%), “it would be too embarrassing” (41.6% vs. 32.5%) and “I would be seen as weak” (46.6% vs. 34.2%). These differences resulted in significantly raised odds ratios (ranging from 1.48 to 2.11), all of which remained significant following adjustment for Service (odds ratios ranging from 1.55 to 2.23).

**Table 3:** Questionnaire Type (Anonymous vs. Identifiable) and Reporting of Barriers to Care and Perceived Social Stigma

	**Identifiable (n = 296)† n (%)**	**Anonymous (n = 315) †† n (%)**	**Chi**^**2**^**statistic (degrees of freedom), P value**	**Odds ratio (95% confidence interval)**	Adjusted* odds ratio (95% confidence interval)
**Barriers to Care**					
It is difficult to schedule an appointment	66 (23.9)	63 (22.0)	0.31 (1), P = 0.580	0.89 (0.60-1.33)	0.95 (0.64-1.41)
It is difficult to get time off	86 (31.1)	108 (36.9)	2.14 (1), P = 0.143	1.30 (0.92-1.84)	1.34 (0.94-1.91)
I don’t trust mental health professionals	36 (12.7)	42 (14.3)	0.34 (1), P = 0.560	1.15 (0.71-1.86)	1.17 (0.73-1.93)
My visit would not remain confidential	69 (24.7)	76 (26.0)	0.13 (1), P = 0.722	1.07 (0.73-1.56)	1.11 (0.76-1.63)
Leaders discourage the use of mental health services	13 (4.6)	27 (9.3)	4.76 (1), P = 0.029	2.11 (1.06-4.18)	2.23 (1.12-4.43)
**Perceived social stigma**					
It would be too embarrassing	93 (32.5)	122 (41.6)	5.16 (1), P = 0.023	1.48 (1.05-2.08)	1.55 (1.10-2.18)
It would affect my career	116 (41.1)	123 (41.8)	0.03 (1), P = 0.864	1.03 (0.74-1.43)	1.08 (0.77-1.51)
Others would have less confidence in me	151 (53.0)	171 (58.0)	1.46 (1), P = 0.227	1.22 (0.88-1.70)	1.29 (0.93-1.80)
My leaders would treat me differently	152 (53.7)	169 (57.3)	0.75 (1), P = 0.387	1.16 (0.83-1.61)	1.21 (0.87-1.69)
My leaders would blame me	67 (23.5)	74 (25.3)	0.26 (1), P = 0.608	1.10 (0.76-1.62)	1.18 (0.81-1.74)
I would be seen as weak	95 (34.2)	136 (46.6)	9.09 (1), P = 0.003	1.68 (1.20-2.35)	1.78 (1.26-2.50)

† n varied from 276 to 286 due to missing data; †† n varied from 287 to 295 due to missing data; *Adjusted for Service.

## Discussion

We showed that there was a statistically significant effect on the reporting of sub-threshold and probable PTSD (but not common mental disorders) and three (out of eleven) stigmatizing beliefs when using an anonymous compared to identifiable questionnaire. These findings could be interpreted in two ways. The first explanation is that there is an underreporting of symptoms and stigmatizing beliefs among those who completed the identifiable questionnaire because they are concerned about the consequences of disclosure and further stigma. The second explanation is that those completing the anonymous survey over report their own symptoms and stigmatizing beliefs. Since there is no obvious benefit of over reporting symptoms or beliefs in an anonymous questionnaire, we consider that the first explanation is the most plausible. However, this can only be confirmed in a linked qualitative study or clinical follow-up assessment. We note that other studies have found similar differences in the reporting of PTSD symptoms among military personnel [2,10-12].

Why we find an association with probable PTSD and not common mental disorders is puzzling. Perhaps military personnel perceive a traumatic stress disorder as being more indicative of weakness than succumbing to the varied and numerous non-traumatic stressors which occur during deployment.

There has been minimal research on the “honesty” of reporting among military personnel. However, general population surveys have shown that anonymous surveys resulted in increased reporting of eating disorder symptoms [13], while there were no differences in reporting of postpartum mood symptoms [14].

Of particular interest is the implications of our results, and how these will differ according to context. The prevalence of probable PTSD in the UK Armed Forces have remained low, at around 4%, since the start of operations in Iraq and Afghanistan [15,16]. This means that the absolute differences between the anonymous and identifiable versions of the questionnaire are not substantial, even though the odds ratio is large. However, one should be cautious of generalizing this finding (based on a military population with a low prevalence of probable PTSD) to a population with a higher prevalence of probable PTSD (for example, the US military among whom PTSD prevalence (post-deployment) is approximately 13%-19% [3,17]). Exactly the same odds ratio would then have a substantial impact on the success of any screening programmed.

We are aware that alternate allocation is a less than ideal form of allocation concealment. Alternate allocation is not a random process and could be open to bias by the assigning clinician [18]. However, the survey took place in an active war zone, which imposes its own practical constraints. The survey team, who were present throughout the time in which personnel were completing the questionnaire, did not observe any exchange of forms or anything to indicate that the alternate allocation was being violated. Given that the only difference between the two questionnaires was whether or not there was a space for name, address and Service number, it seems unlikely that any deviation from this process would have happened. Further, the two groups were similar, in general, on all socio-demographics, military characteristics and combat experiences. A truly randomized study in an operational environment would not have been possible, thus we believe the current design, although not ideal, was the best available in the conditions and was unlikely to have led to bias.

Given the potential difference in the prevalence of probable PTSD, it is fair to ask why use identifiable (but confidential) surveys at all? One reason is because identifiable (but confidential) surveys have the considerable advantage of permitting truly longitudinal data collection. Also, in the chaotic environment of a war zone where potential participants and the survey team moved frequently, identifiable surveys help prevent duplication. There is, therefore, a tradeoff between full anonymisation, used by Hoge et al [3], but not in our otherwise similar study [16]. Hotopf et al [16] were able to prospectively follow up the same people [15], whilst the anonymised study was only able to look at time trends [3]. However, in a UK-based study the differences between anonymised and identifiable data collection are acceptable, but the same results in a setting with a higher prevalence of PTSD would suggest considerable underreporting of symptoms and thus limit the utility of this approach.

We have done several analyses, hence increasing the possibility of associations arising by chance. We have not made adjustments for multiple comparisons in line with the recommendations of Rothman [19].

## Conclusion

We found a significant effect on the reporting of sub-threshold and probable PTSD and certain stigmatizing beliefs (but not common mental disorders) when using an anonymous compared to identifiable questionnaire, with the anonymous questionnaire resulting in a higher prevalence of probable PTSD and certain stigmatizing beliefs. This result was most likely due to an underreporting of symptoms and stigma among those who completed the identifiable questionnaire. These results suggest that researchers need to weigh up the balance between full anonymisation against the use of non-anonymised but confidential survey methods, which permit future follow up. Clinicians need to consider the background prevalence of the disorder being screened – in this study the differences between anonymised and identifiable data collection were probably acceptable, but the same results in a setting with a higher prevalence of PTSD would suggest considerable underreporting of symptoms.

## Abbreviations

GHQ: General Health Questionnaire; MoD: Ministry of Defence; OMHNE: Operational Mental Health Needs Assessment; PCL-C: Post traumatic stress disorder Checklist Civilian Version; PDHA: Post-Deployment Health Assessment; PDHRA: Post-Deployment Health Re-Assessment; PTSD: Post traumatic stress disorder; UK: United Kingdom; US: United States. Neil Greenberg and Simon Wessely are joint last authors

## Competing interests

Neil Greenberg is a full-time member and Norman Jones is a full-time Reserve member of the UK Armed Forces, both are currently seconded to King’s College London. Nicola T Fear, Simon Wessely and Rachel Seddon are (or were formally) employed by the Academic Centre for Defence Mental Health, based at King’s College London which receives funding from the UK Ministry of Defence. Simon Wessely is also honorary civilian consultant advisor in psychiatry to the British Army and a trustee of Combat Stress, a UK charity that provides service and support for veterans with mental health problems.

## Authors’ contributions

All authors listed participated sufficiently in the work to take responsibility for the content. NTF, the corresponding author, has access to all data from the study, both what is reported and what is unreported, and NTF undertook all the analyses presented. RS assisted in the generation of the manuscript. NJ participated in the design of the statistical strategy and the generation of the manuscript. NG was involved in the design of the study, undertook data collection and commented extensively on earlier drafts of the manuscript. SW was involved in the design of the study, the analytical strategy and commented extensively on the manuscript. All authors have seen and approved the final version of this manuscript.

## Pre-publication history

The pre-publication history for this paper can be accessed here:

http://www.biomedcentral.com/1471-2458/12/797/prepub
